# Clinical application of compound Glycyrrhizin tablets in the treatment of patients with Simplex Henoch-Schonlein Purpura and its effect on immune function

**DOI:** 10.12669/pjms.38.1.4609

**Published:** 2022

**Authors:** Chao Li, Zhi-bin Wang

**Affiliations:** 1 Chao Li, Department of Hematology, Beijing Aerospace General Hospital, Beijing, 100076, P.R. China; 2 Zhi-bin Wang^2^ Department of Hematology, Baoding First Central Hospital, Baoding, Hebei, 071000, P.R.China

**Keywords:** Compound Glycyrrhizin, Henoch-Schonlein purpura, Lymphocyte subsets

## Abstract

**Objectives:**

To investigate the curative effect of Compound Glycyrrhizin Tablets in the adjuvant treatment of simplex Henoch-Schonlein purpura and its influence in improving immune function.

**Methods:**

In this retrospective study design was used in this study. Eighty newly diagnosed patients with purpura simplex who visited the outpatient department of Baoding First Central Hospital from June 2017 to February 2020 were included. They were randomly divided into treatment group and control group. The two groups were provided with the same conventional comprehensive treatment. Patients in the treatment group received oral administration of Compound Glycyrrhizin Tablets on the basis of conventional treatment. The clinical efficacy of the treatment group and the control group were compared according to the time and effect of purpura regression, followed by the comparison of changes of T cell subsets before and after treatment.

**Results:**

The total effective rate of the treatment group was 92.5%, which was higher than that of the control group (77.5%) (P < 0.05). The purpura subsidence time of effective patients in treatment group was shorter than that in control group (P < 0.05). There was no significant difference in lymphocyte subsets between the treatment group and the control group before treatment. After treatment, the proportion of CD4+ cells and CD4+/CD8+ cells in the treatment group were obviously higher than that in the control group, and the count of CD8+ cells was evidently lower than that in the control group (P < 0.05).

**Conclusions:**

Compound Glycyrrhizin is effective in the adjuvant treatment of simplex Henoch-Schonlein purpura without obvious adverse reactions, which is valuable for clinical application as an adjuvant.

## INTRODUCTION

Henoch-Schonlein purpura (HSP) is one of the common allergic diseases with extensive small-vessel vasculitis as its basic pathology. Its primary manifestations involve characteristic skin rash, accompanied frequently by gastrointestinal bleeding, renal impairment, abdominal pain, joint pain, etc. Furthermore, there is no consensus yet in the etiology of HSP, which can be induced by the occurrence of allergic reactions to infection, food, drugs, environment and other multiple factors. It can occur at any age, mostly in adolescents, and its incidence peaks in spring and autumn.^1^-^3^ So far, there is an absence of complete clarification of its pathogenesis. According to previous research, immune dysfunction plays an important role in the occurrence and development of diseases.^4^ In addition, there is currently no effective specific method for the treatment of HSP, among which hormone therapy is the major choice, yet with obvious side effects.^5^ Compound Glycyrrhizin Tablet is a compound preparation composed of glycyrrhizin, monoammonium glycyrrhizinate, glycine and methionine. It is a plant extract of glycosides, with the function of immunoregulation. It can treat inflammatory skin diseases associated with T cells such as psoriasis, leukoderma and lichen planus by regulating T cell activation.^6^ Accordingly, in this study, Compound Glycyrrhizin Tablets were used for the adjuvant treatment of simplex HSP, with the purpose to observe its therapeutic effect and impact on the change of lymphocyte subsets.

## METHODS

### General Data:

The subjects of the study were 80 patients with simplex HSP who were admitted to the Outpatient Department of Hematology in Boding First Central Hospital from June 2017 to February 2020. All patients were included due to the first attack of this disease, and met the relevant diagnostic criteria of HSP.^7^ Of the enrolled 80 cases, there were 43 males and 37 females, with an average age of 15.3±1.2 years old (5~36 years old). The average time from onset to the first outpatient visit was 5.7±1.5d days (3~10 d). All patients were randomly divided into the treatment group and the control group, with 40 cases in each group. Among them, there were 21 males and 19 females in the treatment group, with an average age of 15.7±1.9 years old (range, 5-36 years old), and the time from onset to the first outpatient visit of 5.9±1.2d days (range, 3~10d). In addition, 22 males and 18 females were grouped in the control group, with an average age of 15.1±1.7 years old (range, 5-36 years old), and the time from onset to the first outpatient visit of5.4±1.4d days (range, 3~10d). There was no significant difference in general data such as gender, age and course of disease between the two groups (P > 0.05).

### Ethical approval:

The study was approved by the Institutional Ethics Committee of Baoding First Central Hospital on September 10, 2019(No.: 2012-KY-027), and written informed consent was obtained from all participants

### Inclusion Criteria:

1. Patients who met the diagnostic criteria of HSP.
2. Patients who had no fever, abdominal pain, joint pain and other symptoms except skin purpura.
3. Patients who had no abnormality in routine blood test, routine urine test, routine stool test, liver and kidney function test, Antinuclear Antibodies profile examination, etc.

All patients were required to stay in bed at home, keep a light diet, avoid contact with all suspicious allergens, or hot and cold stimulation, and receive oral medication treatment in the Outpatient Department. Patients in the control group were provided with oral administration of Loratadine, Calcium Preparation, Vitamin C, Compound Rutin and other conventional treatment. Meanwhile, The treatment group was treated with Compound Glycyrrhizin Tablets on the basis of conventional treatment (Manufacturer: Eisai [China] Pharmaceutical Co., Ltd.). As for the therapeutic dosage, it was 25mg three times a day for patients aged ≤ 12 years old, while 50mg three times a day, for those aged > 12 years old.

### Detection Methods:

In the treatment group and the control group, 4ml of fasting blood was collected from each patient in the morning and placed into EDTA anticoagulant tubes for examination. According to the requirement, 10ul monoclonal antibodies (CD45-FITC/CD4-PE/CD8-ECD/CD3-PC5) were added to the numbered tubes. After that, 100ul of anticoagulant were supplemented into the tube, which was then mixed well, stored in dark, and incubated at room temperature for 15-20 min. In the next step, 500ul Beckmann hemolysin OptiLyse C was added and reacted for 10 seconds through vortex mixing. After placement in dark for 10-15 minutes, 300ul of 0.9% sodium chloride was added, shaken and mixed well for detection.

### Criteria for Determining Curative Effect Markedly effective:

All relevant symptoms disappeared after treatment, with no abnormality in related examinations. Effective: Patients showed obvious improvement in the condition after treatment, yet with no complete recovery. Ineffective: There was no improvement in the condition after treatment, or accompanied by the appearance of new symptoms, signs and auxiliary examination abnormalities. Total effective rate= (Cases of markedly effective+Cases of effective)/Total cases×100%.

### Observational Indexes:

The two groups of patients were required to have a weekly outpatient review: To observe the regression of skin purpura and record the regression time; to receive routine urine and stool test every week and check the blood biochemistry every two weeks; to observe adverse drug reactions; to evaluate the curative effect after one month; and to measure T lymphocyte subsets (CD3+, CD4+, CD8+, CD4+/CD8+) by flow cytometry based on the collection of fasting venous blood at admission and the second day after the course of treatment.

### Statistical Analysis:

Statistical software SPSS 21.0 was used for data analysis in this study. The measurement data were expressed as mean ± standard deviation (x ± s), and compared by using t test. Meanwhile, the counting data were expressed as percentage or rate, and Chi-square test was used for comparison between groups. P < 0.05 meant that the difference was statistically significant.

## RESULTS

The total effective rate was 92.5% and 77.7% in the treatment group and the control group, respectively is shown in Table-I. The total effective rate of the treatment group was significantly higher than that of the control group, with statistically significant difference (P < 0.05). The purpura regression time was significantly shorter in the treatment group than that in the control group, and the difference was statistically significant (P < 0.05; Table-II).

**Table-I:** Comparison of clinical curative effect between treatment group and control group [n (%)].

Groups	Markedly effective	Effective	Ineffective	Total effective rate (%)
Treatment group (n = 40)	33 (82.5)	4 (10.0)	3 (7.5)	92.5*
Control group (n = 40)	26 (65.0)	5 (12.5)	9 (22.5)	77.5

Compared with the control group,

* P < 0.05.

**Table-II:** Comparison of purpura regression time in effective patients between two groups (x ± s, d).

Groups	Purpura subsidence time
Treatment group (n=37)	5.3 ± 1.6^*^
Control group (n=31)	7.9 ± 1.3

Compared with the control group,

* P < 0.05.

### Adverse reactions:

There were no cases of adverse reactions related to the use of Compound Glycyrrhizin Tablets in the treatment group. The results of 1-month follow-up revealed that the three patinets had mno improvement howed the expansion of purpura in the treatment group. While in the control group, 9 cases were ineffective, four cases showed that the skin purpura was enlarged, two cases had abdominal pain repeatedly, and three patients showed positive urine protein by routine urine test. All cases with ineffective outcome were admitted to the hospital for further treatment.

Before treatment, there was no significant difference in the count of lymphocyte subsets between the treatment group and the control group (P > 0.05). While after treatment, the proportion of CD4+ cells and CD4+/CD8+ cells was remarkably higher, while the proportion of CD8+ cells was significantly lower in the treatment group than those in the control group, with statistically significant differences (P < 0.05). In addition, there was no significant difference in CD3+ cells between the two groups after treatment (P > 0.05). Corresponding results are shown in Table-III.

**Table-III:** Comparison of serum T lymphocyte subsets between the two groups before and after treatment (x±s).

Time	Groups	CD3+ (%)	CD4+ (%)	CD8+ (%)	CD4+/CD8+
Before treatment	Treatment group(n = 40)	58.71±4.32	31.39±5.26	42.02±8.53	0.74±0.21
	Control group (n = 40)	58.13±4.49	31.71±5.28	42.13±9.16	0.73±0.23
	P value	0.883	0.935	0.986	0.957
After treatment	Treatment group (n=37)	72.71±6.32	44.71±3.28	30.43±4.14	1.49±0.42
	Control group (n=31)	68.13±5.49	37.76±3.71	34.43±4.63	1.21±0.49
	P value	0.494	0.02	0.013	0.015

## DISCUSSION

HSP is a disease with vascular allergy as its pathological basis. It has been documented that immune factors are involved significantly in the occurrence and development of the disease.^8^ It is generally considered that the normal immune response of human body depends on various immune cells, among which T cells occupy the most critical position in the process of active immunity.^9^ T cells interact with or restrict each other to form an appropriate immune response. Meanwhile, T lymphocytes can be divided into different subsets according to their various composition and biological functions. CD3+ is the surface marker of all T lymphocytes. To be specific, the proposed subsets can be sub-classified into helper/inducible T lymphocytes (Th) and suppressor/killer T lymphocytes (Ts), namely, CD4+ T cells and CD8+ T cells. In terms of corresponding functions, CD4+T cells can promote B cells to secrete antibodies and regulate the activation of other T cells.^10^-^12^ Simultaneously, CD8+T are identified to be the primary cytotoxic effector cells, which act directly on target cells and exert an inhibitory role in immune function. Under normal circumstances, a stable and balanced immune cell network can be formed on the basis of interaction and restriction between CD4+ T cells and CD8+ T cells, which can be involved in regulating normal immune function and maintaining immune homeostasis. However, the imbalance between them may result in immune dysfunction *in vivo* and induce the occurrence of diseases consequently.^13^ The onset of HSP was associated with an imbalance of Th1/Th2 and Th2 preponderance activation, leading to increased secretion of Th2 cytokines. Then, the immune active cells are stimulated to produce inflammatory factors, resulting in structural changes of vascular endothelial cells. After vascular endothelial cell injury, neutrophils are activated to produce a large number of reactive oxygen species (ROS), which further accelerates the progression of HSP.^14^

Concerning the treatment of HSP, there is still no specific drug for the treatment so far, and conventional treatment is the major choice, including anti-allergy, improving vascular permeability, symptomatic therapy, etc. However, patients may have different responses to treatment.^15^ Prior research evidence has documented that Compound Glycyrrhizin has anti-inflammatory, anti-allergic, antiviral and immunomodulatory effects.^16^ Its pharmacological action is generally exerted through the following approaches. Specifically and firstly, it can directly bind to phospholipase A2, the promoter of arachidonic acid metabolism pathway, which can further hinder its phosphorylation selectively and thus inhibit its activation. Significantly, the blockage of arachidonic acid metabolism at the initial stage can restrict the release of prostaglandins, leukotrienes and other inflammatory mediators. Besides, it can also suppress the activation of complement by inhibiting the C2 level in the classical complement pathway, hence interfering with the release of leukocytes and playing a dual role in preventing inflammation and allergy. Secondly, by inhibiting the destruction of adrenal corticosteroids, Compound Glycyrrhizin can retard the metabolism of glucocorticoids to enhance the physiological effect of glucocorticoids, thereby alleviating inflammatory exudation and small vessel inflammation. In such a way, it exhibits a hormone-like effect and can also avoid hormone-like adverse reactions importantly. Thirdly, it can inhibit the synthesis of immunoglobulin directly or indirectly by altering the distribution of lymphocyte subsets. Consequently, the process may reduce the production of immune complexes, thus suppressing immune response.^17^-^20^ Fourthly, Compound Glycyrrhizin also has a role in weakening the inflammatory reaction by reducing oxygen radicals *in vivo* to alleviate the damage of superoxide to blood vessels. In recent decades, the application of Compound Glycyrrhizin Tablets has achieved a relatively satisfactory outcome in the treatment of urticaria, psoriasis, alopecia areata and various other immune related diseases.^21^-^22^

In our study, outpatient treatment using Compound Glycyrrhizin Tablets obtained satisfactory clinical results in the adjuvant treatment of simplex HSP. The total effective rate was 92.5% in the treatment group, and the purpura regression time was 5.3 ± 1.6 days in effective patients. Corresponding difference was statistically significant when compared with the control group. Previous studies have also shown that the treatment regimen of compound glycyrrhizin tablets combined with glucocorticoids can significantly reduce the levels of febrile inflammatory factors in peripheral blood of patients with HSP without renal injury.^23^ At the same time, 3 ineffective patients in the treatment group had no renal damage, while three of nine had no improvement in the control group had positive urine protein. Of course, owing to a limited sample size of the present study, it still remains unclear whether it is suggested that Compound Glycyrrhizin can reduce the incidence of renal damage in HSP. There is a need to further observe the curative effect based on a larger sample size and expanded scope of research. In addition, as indicated in our study, the treatment group was superior to the control group in improving serum T lymphocyte subsets. Following the use of Compound Glycyrrhizin, those patients with simplex HSP had obviously higher count of CD4+T cells while much lower count of CD8+T than those with conventional treatment in the control group. Collectively, findings in our study support that Compound Glycyrrhizin can improve the immune function of patients with simplex HSP.

### Limitations of this study:

The number of subjects included in this study is limited, so the conclusions drawn may not be very convincing. In addition, the subjects of this study are outpatients, so there may be some non-response bias in the follow-up, which may have some influence on the results of the study.

## CONCLUSIONS

Outpatient use of Compound Glycyrrhizin Tablets has an improved curative effect in the treatment of patients with simplex HSP. It can accelerate purpura regression, shorten the course of treatment, reduce the hospitalization rate, and have no obvious adverse drug reactions. Therefore, Compound Glycyrrhizin Tablets is useful for clinical application as an adjuvant therapy for patients with HSP.

### Authors’ Contributions:

**CLi and ZW** designed this study and prepared this manuscript, and are responsible and accountable for the accuracy or integrity of the work.

**CL** collected and analyzed clinical data.

**ZW** significantly revised this manuscript.
